# Validation of the American Joint Committee on Cancer Eighth Edition Staging of Patients With Metastatic Cutaneous Melanoma Treated With Immune Checkpoint Inhibitors

**DOI:** 10.1001/jamanetworkopen.2021.0980

**Published:** 2021-03-09

**Authors:** Jessica J. Waninger, Vincent T. Ma, Sara Journey, Jeremy Skvarce, Zoey Chopra, Alangoya Tezel, Alex K. Bryant, Charles Mayo, Yilun Sun, Kamya Sankar, Nithya Ramnath, Christopher Lao, Jeremy B. Sussman, Leslie Fecher, Ajjai Alva, Michael D. Green

**Affiliations:** 1 University of Michigan Medical School, University of Michigan, Ann Arbor; 2 Department of Cellular and Molecular Biology, University of Michigan, Ann Arbor; 3 Michigan Center for Translational Pathology, University of Michigan, Ann Arbor; 4 Division of Hematology Oncology, Department of Internal Medicine, University of Michigan, Ann Arbor; 5 Department of Radiation Oncology, University of Michigan, Ann Arbor; 6 Department of Biostatistics, University of Michigan, Ann Arbor; 7 Rogel Cancer Center, University of Michigan, Ann Arbor; 8 Department of Hematology Oncology, Veterans Affairs Ann Arbor Healthcare System, Ann Arbor, Michigan; 9 Department of Medicine, Veterans Affairs Ann Arbor Healthcare System, Ann Arbor, Michigan; 10 Center for Clinical Management Research, Veterans Affairs Ann Arbor Healthcare System, Ann Arbor, Michigan; 11 Institute for Healthcare Policy and Innovation, University of Michigan, Ann Arbor; 12 Department of Radiation Oncology, Veterans Affairs Ann Arbor Healthcare System, Ann Arbor, Michigan

## Abstract

**Importance:**

Immune checkpoint inhibitors (ICIs) have transformed the survival of patients with metastatic melanoma. Patient prognosis is reflected by the American Joint Committee on Cancer (AJCC) staging system; however, it is unknown whether the metastatic (M) stage categories for cutaneous melanoma remain informative of prognosis in patients who have received ICIs.

**Objectives:**

To evaluate the outcomes of patients with metastatic cutaneous melanoma based on the M stage category from the AJCC eighth edition and to determine whether these designations continue to inform the prognosis of patients who have received ICIs.

**Design, Setting, and Participants:**

This cohort study included patients with metastatic cutaneous melanoma who were treated between August 2006 and August 2019 at the University of Michigan. The estimated median follow-up time was 35.5 months. Patient data were collected via the electronic medical record system. Critical findings were externally validated in a multicenter nationwide cohort of patients treated within the Veterans Affairs health care system. Data analysis was conducted from February 2020 to January 2021.

**Exposures:**

All patients were treated with dual-agent concurrent ipilimumab and nivolumab followed by maintenance nivolumab or single-agent ipilimumab, nivolumab, or pembrolizumab therapy. Patients were staged using the AJCC eighth edition.

**Main Outcomes and Measures:**

Univariable and multivariable analyses were used to assess the prognostic value of predefined clinicopathologic baseline factors on survival.

**Results:**

In a discovery cohort of 357 patients (mean [SD] age, 62.6 [14.2] years; 254 [71.1%] men) with metastatic cutaneous melanoma treated with ICIs, the M category in the AJCC eighth edition showed limited prognostic stratification by both univariable and multivariable analyses. The presence of liver metastases and elevated levels of serum lactate dehydrogenase (LDH) offered superior prognostic separation compared with the M category (liver metastases: hazard ratio, 2.22; 95% CI, 1.48-3.33; *P* < .001; elevated serum LDH: hazard ratio, 1.73; 95% CI, 1.16-2.58; *P* = .007). An updated staging system based on these factors was externally validated in a cohort of 652 patients (mean [SD] age, 67.9 [11.6] years; 630 [96.6%] men), with patients without liver metastases or elevated LDH levels having the longest survival (median overall survival, 30.7 months).

**Conclusions and Relevance:**

This study found that the AJCC eighth edition M category was poorly reflective of prognosis in patients receiving ICIs. Future staging systems could consider emphasizing the presence of liver metastases and elevated LDH levels. Additional studies are needed to confirm the importance of these and other prognostic biomarkers.

## Key Points

**Question:**

Is the American Joint Committee on Cancer (AJCC) eighth edition staging of metastatic cutaneous melanoma prognostic in the era of immune checkpoint inhibition?

**Findings:**

In this cohort study of 357 patients with metastatic cutaneous melanoma treated with immune checkpoint inhibitors, the AJCC eighth edition M staging showed limited prognostic stratification; however, the overall survival rates among patients with liver metastases and those with elevated serum lactate dehydrogenase (LDH) levels were much worse than among patients with metastatic involvement of other organs and those with serum LDH levels in the reference range. The importance of these factors was confirmed in a multi-institutional external validation cohort.

**Meaning:**

These findings suggest that other biomarkers that are associated with overall survival, such as liver metastases and LDH level, should be considered for emphasis in future staging systems.

## Teaser

This cohort study evaluates the outcomes of patients with metastatic cutaneous melanoma based on the M stage category from the American Joint Committee on Cancer (AJCC) eighth edition and determines whether these designations continue to inform the prognosis of patients who have received immune checkpoint inhibitors (ICIs).

## Introduction

Immune checkpoint inhibitors (ICIs) targeting cytotoxic T-lymphocyte-antigen 4 and programed cell death ligand 1 (PD-1) have transformed the outcomes of patients with metastatic cutaneous melanoma. Currently approved ICIs for the treatment of metastatic melanoma include anti–CTLA-4 monotherapy (ipilimumab), anti–PD-1 monotherapy (nivolumab and pembrolizumab), and combination ipilimumab and nivolumab. Additionally, targeted therapies approved for the treatment of *BRAF* V600–altered melanoma include BRAF inhibitors (dabrafenib, vemurafenib, encorafenib) and MEK inhibitors (trametinib, combimetinib, binimetinib). Together, these classes of agents have dramatically improved patient outcomes compared with prior systemic agents, such as cytotoxic chemotherapy and interleukin 2.^1,2^

Staging informs patient-centered discussions on prognosis as well as clinical decision-making. The American Joint Committee on Cancer (AJCC) revised the AJCC melanoma staging manual in 2017, releasing an eighth edition. Updates to the tumor (T) stage included alterations to tumor thickness cutoffs and ulceration status. In the nodal (N) category, new descriptors and more formal stratification were implemented to describe regional nodal and nonnodal disease. Prior to the eighth edition, the metastatic (M) stage included 3 categories based on the anatomic site of disease involvement, as follows: M1a (nonregional lymph nodes and/or skin or soft tissue lesions), M1b (lung involvement), and M1c (other visceral sites of disease). In the eighth edition, the M staging system was updated to include the addition of lactate dehydrogenase (LDH) subcategories for each stratum and the addition of a new M1d designation for patients with central nervous system metastases.^3^ The creation of the M1d category was based on expert clinical assessment given that patients with central nervous system metastases are often excluded from clinical trials. The melanoma expert panel acknowledged that amending the M stage further was premature, and additional revisions are anticipated.

To gain insight into how the routine use of ICIs has affected patient prognosis, we conducted a retrospective analysis of patients with metastatic cutaneous melanoma who were treated with either single or dual immunotherapeutic agents. LDH level has been shown to be a prognostic variable in a variety of cancers, especially melanoma,^4,5,6^ and to modify antitumor responses.^7,8^ Furthermore, emerging evidence suggests that liver metastases influence patient response to ICI.^9,10^ Therefore, we examined the prognostic importance of these variables.

## Methods

### Clinical Cohorts

We performed a single-center, retrospective analysis of patients with metastatic cutaneous melanoma who were treated between August 2006 and August 2019 (with most treated 2014-2019) using the AJCC eighth edition. All patients were treated with standard-of-care ipilimumab and nivolumab combination therapy (concurrent) or single-agent ipilimumab, nivolumab, or pembrolizumab therapy. Patients and data were collected via the University of Michigan electronic medical record system. Patient data were extracted manually as well as from the Michigan Radiation Oncology Analytics Resource, a custom system.^11^ Baseline patient demographic information, including age, sex, M category designation per the AJCC eighth edition at the start of immunotherapy, *BRAF *status, and ICI agent, were collected. Collection included the following prognostic factors: Eastern Cooperative Oncology Group (ECOG) performance status; serum LDH level at the time of metastatic disease diagnosis, with the upper reference limit (ie, 240 U/L [to convert to microkatals per liter, multiply by 0.0167]) used as a cutoff for LDH elevation; presence or absence of liver and brain metastases at time of ICI initiation; number of prior lines of therapy; and total number of metastatic disease sites. The end points analyzed included overall survival (OS) or progression-free survival (PFS) based on designated M category. Patients with missing values pertinent to the specific analysis were excluded. Less than 5% of patients were lost to follow-up.

For external validation of critical findings, we identified a national cohort of US veterans with biopsy-proven metastatic cutaneous melanoma treated with ICIs between 2010 and 2019 using the Veteran Affairs Informatics and Computing Infrastructure (VINCI). VINCI is a comprehensive informatics platform that allows researchers access to patient-level electronic health record information and administrative data for all veterans within the Veteran Affairs (VA) health care system. VINCI incorporates tumor registry data uploaded from individual VA sites; these data are gathered at individual VA medical centers by trained registrars according to standard protocols issued from the American College of Surgeons. Tumor registry data were supplemented with intravenous infusion administration records for ipilimumab, pembrolizumab, and/or nivolumab to identify patients. Baseline clinical, pathologic, and radiographic characteristics at the start of immunotherapy were collected. OS was analyzed as previously described, and date of death or last follow-up was collected from internal VA vital status data. Patients with missing values pertinent to the specific analysis were excluded. The VA health care system provides nationwide care, and all hospitals were queried to minimize patients lost to follow up.

All clinical records were obtained with the approval of the University of Michigan and VA institutional review boards. Most patients were deceased, and following institutional guidelines, the requirement for informed consent was waived. This study followed the Strengthening the Reporting of Observational Studies in Epidemiology (STROBE) reporting guideline.

### Statistical Analysis

Patient date of death (DOD), date of last follow-up, and time to progression were used to generate Kaplan-Meier plots of OS and PFS followed by log-rank tests. For OS analyses, DOD was used as a primary end point. Patients still alive were censored at the time of last follow-up. Time of progression was used as the primary end point for PFS analyses, and DOD was used as the secondary end point for patients without a progression date. Statistical analyses including pairwise comparisons and Cox proportional hazard models were done using Prism version 8 and SPSS statistical software version 27 (IBM Corp). Hazard ratios were calculated using the Mantel-Haenszel method and reported with a 95% CI and a significance cut-off of *P* < .05. All tests were 2-tailed. Royston D statistics were calculated for each prognostic model. Follow-up duration was determined using the reverse Kaplan-Meier method. All analyses were conducted with SAS version 9.4 (SAS Institute).

## Results

We examined a single-institutional cohort of 357 patients with metastatic cutaneous melanoma. The estimated median (95% CI) follow-up time was 35.5 (33.2-37.4) months. Patient demographic information and relevant prognostic variables are summarized in the Table. The mean (SD) age of patients was 62.6 (14.2) years, and most were men (254 [71.1%]), with a preserved performance status (ie, ECOG score 0-1; 338 [94.7%]). A slight majority of patients had *BRAF* V600 wild-type (WT) tumors (186 [52.1%]). Overall, 305 patients (85.4%) received ICI as first-line treatment, with 52 (14.6%) receiving ICIs as second-line or greater treatment. Both single agent ICI (240 [67.2%]) and combination ipilimumab and nivolumab (117 [32.8%]) were commonly prescribed. A total of 297 patients (83.2%) had more than 1 site of metastasis, and the median (interquartile range [IQR]) number of metastatic sites was 3 (2-5). Nearly one-third of patients (108 [30.3%]) had baseline involvement of the liver, and more than one-quarter (95 [26.6%]) had baseline involvement of the brain at time of ICI initiation. Overall, 92 (25.8%) had LDH values greater than the upper reference limit of normal (ie, 240 U/L).

**Table.** Baseline Patient and Disease Characteristics

Factor	Patients, No. (%) (N = 357)
Age at ICI start	
Mean (SD)	62.6 (14.2)
Median (IQR)	63.7 (53.7-72.5)
Sex	
Women	103 (28.9)
Men	254 (71.1)
*BRAF *status	
WT	186 (52.1)
Variant	151 (42.3)
Unknown	20 (5.6)
ECOG performance score	
0	133 (37.3)
1	205 (57.4)
≥2	19 (5.3)
ICI type	
Dual agent^a^	117 (32.8)
Single agent	240 (67.2)
Prior lines of therapy	
0	305 (85.4)
1	42 (11.8)
2	6 (1.7)
3	3 (0.8)
4	1 (0.3)
M category at ICI initiation	
M1a	37 (10.4)
M1b	55 (15.4)
M1c	166 (46.5)
M1d	99 (27.7)
Metastatic sites, No.	
1	60 (16.8)
≥2	297 (83.2)
Presence of liver metastases	
No	249 (69.7)
Yes	108 (30.3)
Presence of brain metastases	
No	223 (62.5)
Yes	99 (27.7)
Unknown	35 (9.8)
LDH level	
Elevated, ≥240 IU/L	92 (25.8)
WRL, ≤240 IU/L	242 (67.8)
Unknown	23 (6.4)

Abbreviations: ECOG, Eastern Cooperative Oncology Group; ICI, immune checkpoint inhibitors; IQR, interquartile range; LDH, lactate dehydrogenase; WRL, with reference limit; WT, wild type.

SI conversion: To convert LDH to microkatals per liter, multiply by 0.0167.

^a^ Dual-agent ICI indicates concurrent ipilimumab and nivolumab.

**Figure 1.**
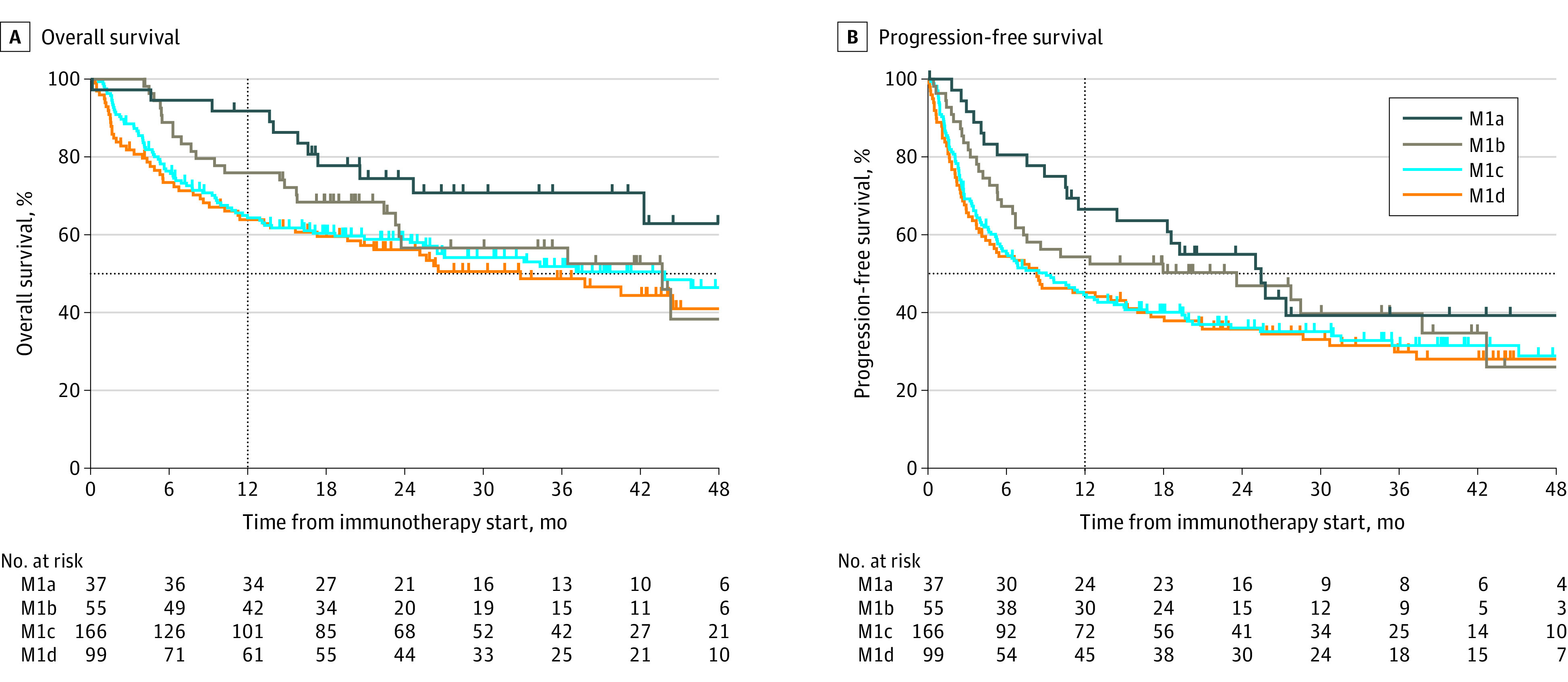
Univariable Analysis of Patients With Metastatic Cutaneous Melanoma Treated With Immune Checkpoint Inhibitors by American Joint Committee on Cancer Eighth Edition Metastasis (M) Staging M1a indicates patients with nonregional lymph nodes and/or skin or soft tissue lesions; M1b, patients with lung metastases; M1c, patients with all other visceral sites of disease (including liver involvement); M1d, patients with central nervous system metastases. Vertical line marks 1-year survival.

**Figure 2.**
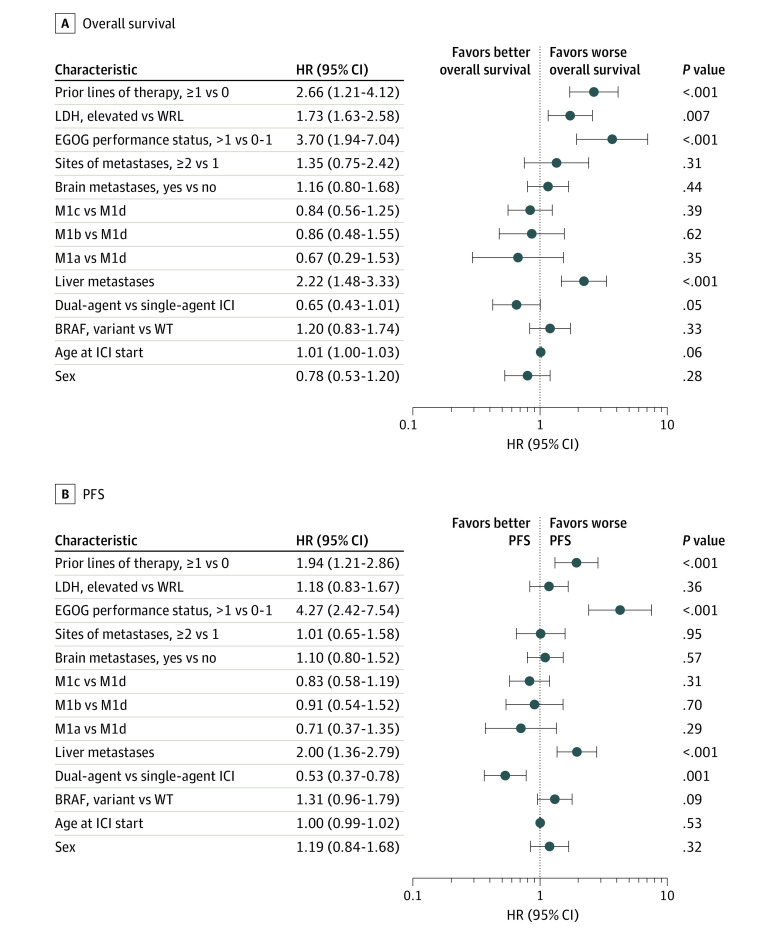
Multivariable Analysis of Patients With Metastatic Cutaneous Melanoma Treated With Immune Checkpoint Inhibitors (ICIs) Single-agent ICI indicates programmed death cell ligand 1 or cytotoxic T-lymphocyte antigen 4 inhibition, while dual-agent ICI indicates concurrent ipilimumab and nivolumab. ECOG indicates Eastern Cooperative Oncology Group; HR, hazard ratio; LDH, lactate dehydrogenase; M1a, patients with nonregional lymph nodes and/or skin or soft tissue lesions; M1b, patients with lung metastases; M1c, patients with all other visceral sites of disease (including liver involvement); M1d, patients with central nervous system metastases; PFS, progression-free survival; WRL, within reference limits (ie, ≤240 U/L [to convert to microkatals per liter, multiply by 0.0167]); WT, wild type.

To understand the prognostic value of the AJCC eighth edition M categories in patients receiving ICIs, we assessed patient OS and PFS stratified by M category at the time of ICI initiation (Figure 1; eTable 1 and eTable 2 in the Supplement). The model showed poor prognostic separation between each M category (OS: D statistic, 1.4; 95% CI, 1.1-1.8; PFS: D statistic, 1.3; 95% CI, 1.0-1.6). Model statistics computed for patient stage at metastatic disease diagnosis also suggested the limited prognostic utility of the current staging system (OS: D statistic, 1.3; 95% CI, 0.99-1.7; PFS: D statistic, 1.2; 95% CI, 0.97-1.5) (eTable 3 in the Supplement). Only patients designated as having stage M1a disease at the start of ICI had superior outcomes compared with patients with M1c or M1d disease but not those designated as having M1b disease (Figure 2; eTable 2 in the Supplement). Patients staged as having M1c disease had a similar OS and PFS as those with M1d disease (OS: hazard ratio [HR], 0.91; 95% CI, 0.64-1.30; *P* = .61; PFS: HR, 0.92; 95% CI, 0.68-1.24; *P* = .57). Lack of prognostic separation between M categories for both OS and PFS (Figure 2A and Figure 2B), including M1c and M1d, was persistent after adjusting for prognostic variables such as age, sex, LDH level, *BRAF* status, ECOG performance status, receipt of single-agent or dual-agent ICI, prior lines of therapy, presence or absence of liver or brain metastases, and total number of metastatic sites. Furthermore, the presence of brain metastases had no significant association with survival outcomes in patients receiving ICI (eFigure 1A and eFigure 1B in the Supplement) (univariable analysis, OS: HR, 0.79; 95% CI, 0.56-1.12; *P* = .18; PFS: HR, 0.81; 95% CI, 0.61-1.09; *P* = .16; multivariable analysis, OS: HR, 1.16; 95% CI, 0.80-1.68; *P* = .44; PFS: HR, 1.10; 95% CI, 0.80-1.52; *P* = .57). Given that 52 patients (14.6%) in our cohort received prior therapies, we performed a subset analysis including only patients who received first-line ICI. Similar to the findings described previously, there was no association between designated M categories and PFS or OS (eFigure 2A, eFigure 2B, and eTable 4 in the Supplement). Owing to the importance of *BRAF* V600 alteration status as a biomarker of response, we compared the survival outcomes between patients with *BRAF* WT tumors and those with *BRAF *variant tumors in our cohort and found that *BRAF* status was significantly associated with poor PFS (HR, 0.73; 95%CI, 0.55-0.96; *P* = .02) but not OS (HR, 0.79; 95% CI, 0.57-1.09; *P* = .15) in univariable analysis (eFigure 3A and eFigure 3B in the Supplement). In multivariable modeling, *BRAF* status was not significantly associated with OS (HR, 1.20; 95% CI, 0.83-1.74; *P* = .33) or PFS (HR, 1.31; 95% CI, 0.96-1.79; *P* = .09) (Figure 2A and Figure 2B).

**Figure 3.**
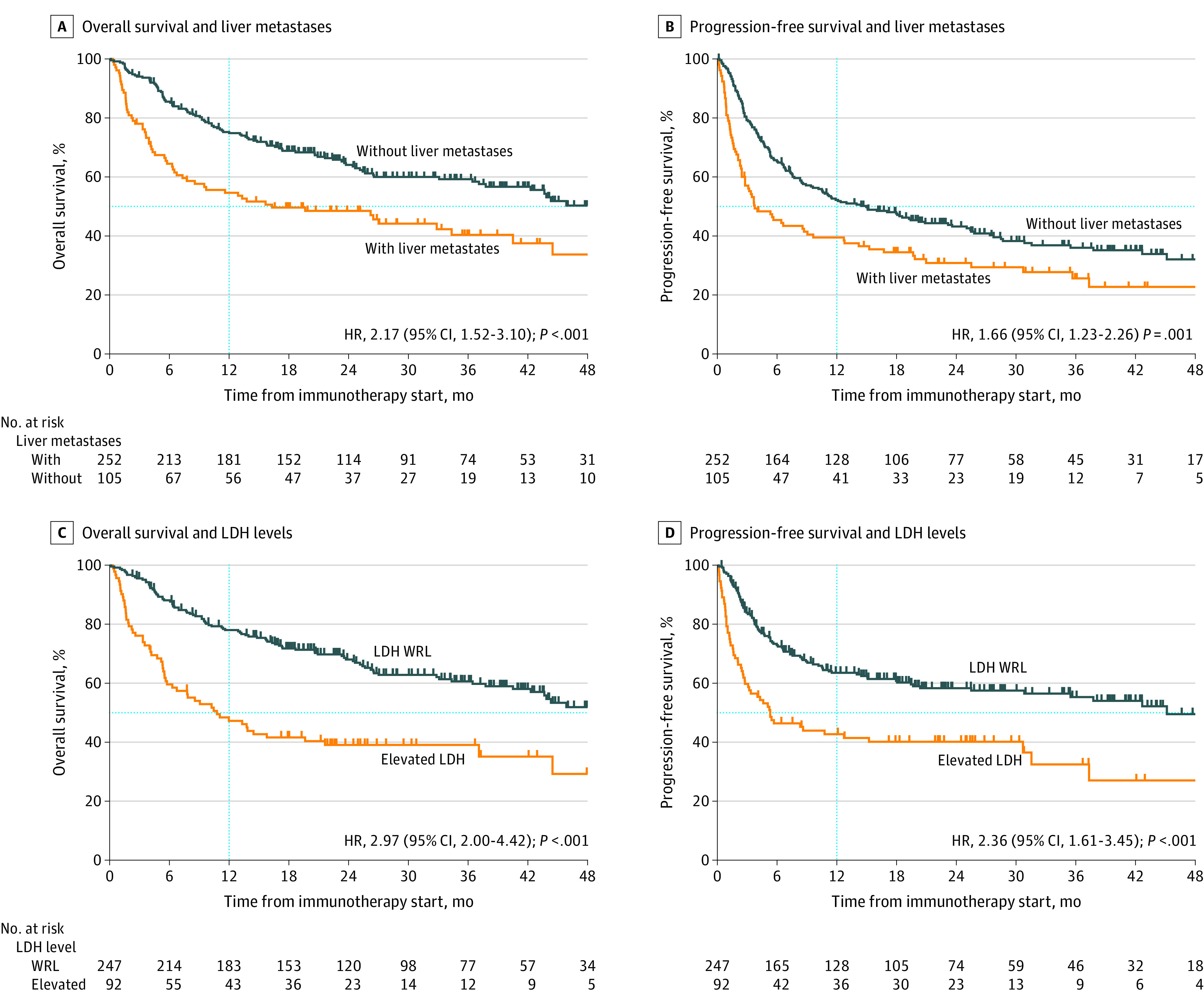
Association of Liver Metastases and Lactate Dehydrogenase (LDH) Levels With Survival Vertical dotted line marks 1-year survival. HR indicates hazard ratio; WRL, within reference limit (ie, ≤240 U/L [to convert to microkatals per liter, multiply by 0.0167]).

We next sought to identify clinically relevant prognostic biomarkers for patients with metastatic cutaneous melanoma receiving ICI. Recent data suggest that liver metastases have a negative prognostic association with immunotherapy response and survival outcomes in patients with melanoma.^9,10^ Consistent with previous work, we saw that the presence of liver metastasis was associated with worse survival outcomes in patients treated with ICIs (OS: HR, 2.17; 95% CI, 1.52-3.10; *P* < .001; PFS: HR, 1.66; 95% CI, 1.23-2.26; *P* = .001) (Figure 3A and 3B). Furthermore, we assessed the relative contribution of liver metastases to patients with both M1c and M1d disease. We found that patients with liver metastases had shorter PFS and OS than patients without liver involvement regardless of AJCC M category (eFigure 4 in the Supplement). Median (IQR) OS was 16.3 (3.5-28.8) months for patients with M1c disease and liver metastases vs 56.5 (10.8-62.2) months for those with M1c disease and no liver metastases (HR, 0.52; 95% CI, 0.33-0.82; *P* = .004). For patients with M1d disease and liver metastases, median (IQR) OS was 19.6 (3.3-32.9) months vs 62.0 (8.3-63.7) months for patients with M1d and no liver metastases (HR, 0.53; 95% CI, 0.29-0.99; *P* = .045). Although patients with M1c disease and liver involvement had significantly worse PFS than those with M1c disease and no liver involvement (median [IQR] PFS, 3.7 [1.4-19.7] months vs 12.1 [4.3-28.9] months; HR, 0.64; 95% CI, 0.43-0.95; *P* = .03) patients with M1d disease and liver involvement did not have significantly different PFS than those with M1d disease and no liver involvement (median [IQR] PFS, 8.6 [0.7-27.8] months vs 8.3 [2.3-28.6] months; HR, 0.71; 95% CI, 0.42-1.21; *P* = .21) (eTable 5 and eTable 6 in the Supplement). While patients with brain metastases tend to have worse outcomes than those without brain involvement, our data suggested that liver metastases were more significantly associated with survival outcomes in the context of patients treated with ICI.

In our cohort, patients with reference LDH levels (ie, ≤240 U/L) had significantly better survival outcomes than those with elevated levels (OS: HR, 2.97; 95% CI, 2.00-4.42; *P* < .001; PFS: HR, 2.36; 95% CI, 1.61-3.45; *P* < .001) (Figure 3C and 3D). It is thought that elevations in LDH level are reflective of tumor burden; however, we saw only a weak correlation between the number of metastatic sites and LDH level (eFigure 5 in the Supplement).

To understand the combined association of LDH and liver involvement with OS and PFS, we stratified patients by both LDH level and the presence or absence of liver metastases. While both liver metastases and elevated LDH were independently associated with worse survival, the concomitant presence of both factors was associated with worse prognosis (eTable 7 in the Supplement).

**Figure 4.**
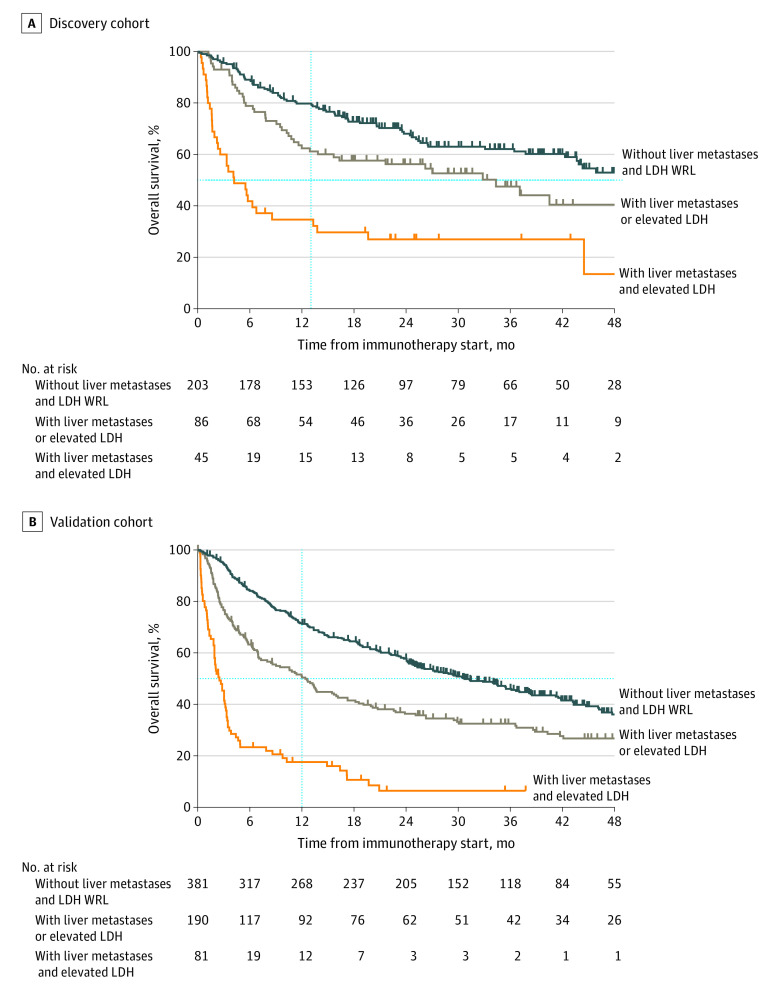
Overall Survival in Discovery and Validation Cohorts by Lactate Dehydrogenase (LDH) Level and Liver Metastases Vertical dotted line marks 1-year survival. WRL indicates within reference limit (ie, ≤240 U/L [to convert to microkatals per liter, multiply by 0.0167]).

Given that both LDH levels and liver involvement appeared to be important prognostic indicators of PFS and OS in patients receiving ICI, we analyzed the possibility of a new staging system in which patients were stratified into the 3 following categories: (1) reference LDH level (WRL LDH) and no liver metastases, (2) elevated LDH level without liver metastases or WRL LDH with liver metastases, and (3) both elevated LDH levels and evidence of liver involvement. The median (IQR) survival for patients with WRL LDH and no liver metastases was greater (OS, 56.5 [15.8-73.8] months; PFS, 18.0 [4.5-28.4] months) than that for patients with either high LDH or the presence of liver metastases (OS, 34.4 [7.7-56.2] months; PFS, 8.5 [2.6-25.5] months), which was much greater than that for patients with both (OS, 4.2 months [1.6-19.5]; and PFS, 2.1 [0.8-17.3] months) (Figure 4A; eFigure 6, eTable 8, and eTable 9 in the Supplement).

To confirm the validity of these findings, we identified a nationwide multicenter cohort of US veterans with metastatic cutaneous melanoma who received ICI. Patient demographic information and relevant prognostic variables are summarized in eTable 10 in the Supplement. Overall, 652 patients were identified, with a mean (SD) age of 67.9 (11.6) years. The estimated median (IQR) follow-up time was 43.1 (30.6-55.6) months. Almost all patients were men (630 [96.6%]), and most received single-agent ICI (563 [86.3%]). In this cohort, patients without liver metastases or elevated LDH levels had the longest survival (median [IQR] OS, 30.7 [9.8-39.7] months) compared with patients with either liver metastases or elevated LDH levels (median [IQR] OS, 12.4 [3.2-31.0] months) (Figure 4B; eTable 11 and eTable 12 in the Supplement). Patients with both liver metastases and elevated LDH levels had the shortest survival (median [IQR] OS, 2.5 [1.1-4.8] months). Together, these results confirm the relative association of both liver metastases and LDH levels with the prognosis of patients with metastatic cutaneous melanoma receiving ICI therapy.

## Discussion

The findings of this study suggest that the AJCC eighth edition staging of metastatic cutaneous melanoma (M1a-d) requires revisions in the era of ICI therapy. We additionally found that the presence of liver metastases at the start of immunotherapy and elevations in LDH level were associated with patient survival outcomes. These findings suggest that a staging model retaining LDH levels and incorporating the liver as a specific anatomic site of involvement may aid in patient prognostication.

While updates from the sixth to the seventh edition of the AJCC melanoma staging system confirmed prognostic separation between M1a (skin, subcutaneous, or nonregional lymph node metastases), M1b (metastasis to the lung), and M1c (any nonpulmonary visceral metastatic site and any patient with elevated LDH level)^12^ categories, the rapid changes in advanced melanoma treatment (including ICI and targeted therapy) at the time of its release limited revisions of the AJCC eighth edition M stage. Although brain metastases are common among solid tumors, especially metastatic melanoma, and can cause debilitating neurologic complications, activity of dual-agent ICI as well as multidisciplinary management of brain metastases have enhanced outcomes of patients with intracranial involvement.^13,14^ Our study illustrated that routine ICI treatment has limited the prognostic stratification of patients using the AJCC eighth edition and that while brain involvement was still a negative prognostic factor, it was not significantly associated with poor outcomes in our cohort.

It has long been recognized that liver involvement is a poor prognostic factor in breast and colorectal cancer.^15,16^ Liver metastases have been shown to be immunosuppressive^17^ and have been associated with a decreased response to PD-1 blockade and shortened OS across tumor types.^9,10,18,19,20^ Our study found that the presence of liver metastases at the start of ICI was significantly associated with patient outcomes.

Another important factor associated with outcomes among patients with metastatic melanoma patient was LDH level, independent of tumor burden.^3,21^ An overwhelming amount of evidence demonstrates that elevated LDH levels are associated with poor outcomes in patients with cancer, especially those with metastatic melanoma,^22^ and more recently, studies have shown that high LDH levels are a poor prognostic indicator in patients receiving immunotherapy.^23^ Preclinical studies have shown that elevations in LDH promote immunosuppression by enhancing the recruitment of T-regulatory cells, inhibiting natural killer (NK) cells, and increasing myeloid-derived suppressor cell frequency.^7,8,24,25^ While many studies suggest that elevated levels of LDH are reflective of tumor burden, we observed minimal correlation between the number of metastatic sites and LDH levels (eFigure 5 in the Supplement). Our data support LDH as an important independent factor in this cohort. When coexistent with liver metastases, elevated LDH levels were associated with even worse outcomes. Our data confirm the continued prognostic relevance of LDH in metastatic cutaneous melanoma, especially for patients treated with ICI.

In the current therapeutic landscape, BRAF/MEK inhibition represents an alternative therapeutic option in patients with *BRAF* V600–altered melanoma. Based on limited studies, the effectiveness of ICI is thought to be independent of BRAF alteration status.^26^ To date, there is a paucity of robust data on the outcomes of patients with *BRAF* V600 alterations treated with ICIs following BRAF-targeted therapy or vice versa.^27^ In our study, we analyzed the contribution of the *BRAF* alteration on patient survival outcomes. We found that patients with *BRAF* alterations had a shorter time to progression but similar OS as patients with *BRAF* WT tumors. Importantly, *BRAF* variant status was no longer significantly associated in the multivariable setting. Prospective studies^28,29^ evaluating the differential outcomes based on *BRAF* variant status are in progress.

### Limitations

This study has limitations. Our cohort contained a heterogenous group of patients, including those with brain metastases, and is reflective of the variability seen in a real-world clinic setting. Important limitations include the retrospective nature and relatively limited sample size, particularly in the M1a and M1b subgroups. In addition, while most patients in our cohort received first-line ICI (85.4%), a small subset (14.6%) received prior therapies. Furthermore, other potential prognostic biomarkers, including tumoral PD-1 and tumor mutation burden, were not collected.^30,31^

## Conclusions

This study showed that elevations in LDH level and metastatic liver involvement in patients with melanoma were associated with prognosis and, therefore, may inform therapeutic decision-making. The prognosis of metastatic cutaneous melanoma has changed drastically in the era of ICI and targeted therapies. Future updates to staging systems are needed for the optimal stratification of patients into clinically relevant prognostic groups. Additional studies will be needed to further confirm our findings and develop novel treatment strategies to enhance the prognosis of patients with metastatic cutaneous melanoma and liver metastases or elevated levels of LDH.

## Notes

Supplement. **eTable 1. **OS and PFS Measures by AJCC Eighth Edition M category **eTable 2. **Pairwise Comparisons of each AJCC Eighth Edition M category **eTable 3.** Royston D Statistic of M Staging Models **eFigure 1. **Kaplan-Meier Analysis of the OS and PFS in Patients With and Without Brain Metastases **eFigure 2. **Kaplan-Meier Analysis of AJCC Eighth Edition M Category at ICI Initiation in Patients Who Received First-Line ICI **eTable 4.** Pairwise Comparisons of Each AJCC Eighth Edition M Category for Patients Who Received First-Line ICI **eFigure 3.** Kaplan-Meier Analysis of Patient OS and PFS by *BRAF* Status **eFigure 4.** Kaplan-Meier Analysis Comparing the OS and PFS of Patients With and Without Liver Metastases **eTable 5. **OS and PFS Measures for Patients With M1c and M1d Disease With and Without Liver Metastases **eTable 6. **Pairwise Comparisons of Patients With M1c and M1d Disease With and Without Liver Metastases **eFigure 5.** Comparison of Number of Metastatic Sites and LDH Level **eTable 7. **Pairwise Univariable Analysis of LDH Stratified by Liver Involvement **eTable 8. **OS and PFS Measures of Patients in the Proposed Staging Model **eTable 9. **Pairwise Comparisons of Each Stratum in the Proposed Staging Model of Patients With Metastatic Cutaneous Melanoma **eFigure 6. **Kaplan-Meier Analysis of Progression-Free Survival of the Proposed Staging Model of Patients With Metastatic Cutaneous Melanoma Incorporating LDH Levels and Liver Involvement **eTable 10. **Clinical and Disease Characteristics of External Validation Cohort **eTable 11. **OS Measures of External Validation Cohort **eTable 12. **Pairwise Comparisons of Each Stratum in the New Staging Model Using an External Validation Cohort
