# Supplementary material for: Validation of the American Joint Committee on Cancer Eighth Edition Staging of Patients With Metastatic Cutaneous Melanoma Treated With Immune Checkpoint Inhibitors
Source: JAMA Netw Open. 2021 Mar 9;4(3):e210980. doi: 10.1001/jamanetworkopen.2021.0980 (PMC7944385; doi:10.1001/jamanetworkopen.2021.0980)
Supplement: Supplement. — eTable 1. OS and PFS Measures by AJCC Eighth Edition M category eTable 2. Pairwise Comparisons of each AJCC Eighth Edition M category eTable 3. Royston D Statistic of M Staging Models eFigure 1. Kaplan-Meier Analysis of the OS and PFS in Patients With and Without Brain Metastases eFigure 2. Kaplan-Meier Analysis of AJCC Eighth Edition M Category at ICI Initiation in Patients Who Received First-Line ICI eTable 4. Pairwise Comparisons of Each AJCC Eighth Edition M Category for Patients Who Received First-Line ICI eFigure 3. Kaplan-Meier Analysis of Patient OS and PFS by BRAF Status eFigure 4. Kaplan-Meier Analysis Comparing the OS and PFS of Patients With and Without Liver Metastases eTable 5. OS and PFS Measures for Patients With M1c and M1d Disease With and Without Liver Metastases eTable 6. Pairwise Comparisons of Patients With M1c and M1d Disease With and Without Liver Metastases eFigure 5. Comparison of Number of Metastatic Sites and LDH Level eTable 7. Pairwise Univariable Analysis of LDH Stratified by Liver Involvement eTable 8. OS and PFS Measures of Patients in the Proposed Staging Model eTable 9. Pairwise Comparisons of Each Stratum in the Proposed Staging Model of Patients With Metastatic Cutaneous Melanoma eFigure 6. Kaplan-Meier Analysis of Progression-Free Survival of the Proposed Staging Model of Patients With Metastatic Cutaneous Melanoma Incorporating LDH Levels and Liver Involvement eTable 10. Clinical and Disease Characteristics of External Validation Cohort eTable 11. OS Measures of External Validation Cohort eTable 12. Pairwise Comparisons of Each Stratum in the New Staging Model Using an External Validation Cohort [file jamanetwopen-e210980-s001.pdf]

Supplemental Online Content

Waninger JJ, Ma VT, Journey S, et al. Validation of the American Joint Committee on Cancer eighth edition staging of patients with metastatic cutaneous melanoma treated with immune checkpoint inhibitors. *JAMA Netw Open*. 2021;4(3):e210980. doi:10.1001/jamanetworkopen.2021.0980

eTable 1. OS and PFS Measures by AJCC Eighth Edition M category
eTable 2. Pairwise Comparisons of each AJCC Eighth Edition M category
eTable 3. Royston D Statistic of M Staging Models
eFigure 1. Kaplan-Meier Analysis of the OS and PFS in Patients With and Without Brain Metastases
eFigure 2. Kaplan-Meier Analysis of AJCC Eighth Edition M Category at ICI Initiation in Patients Who Received First-Line ICI
eTable 4. Pairwise Comparisons of Each AJCC Eighth Edition M Category for Patients Who Received First-Line ICI
eFigure 3. Kaplan-Meier Analysis of Patient OS and PFS by *BRAF* Status
eFigure 4. Kaplan-Meier Analysis Comparing the OS and PFS of Patients With and Without Liver Metastases
eTable 5. OS and PFS Measures for Patients With M1c and M1d Disease With and Without Liver Metastases
eTable 6. Pairwise Comparisons of Patients With M1c and M1d Disease With and Without Liver Metastases
eFigure 5. Comparison of Number of Metastatic Sites and LDH Level
eTable 7. Pairwise Univariable Analysis of LDH Stratified by Liver Involvement
eTable 8. OS and PFS Measures of Patients in the Proposed Staging Model
eTable 9. Pairwise Comparisons of Each Stratum in the Proposed Staging Model of Patients With Metastatic Cutaneous Melanoma
eFigure 6. Kaplan-Meier Analysis of Progression-Free Survival of the Proposed Staging Model of Patients With Metastatic Cutaneous Melanoma Incorporating LDH Levels and Liver Involvement
eTable 10. Clinical and Disease Characteristics of External Validation Cohort
eTable 11. OS Measures of External Validation Cohort
eTable 12. Pairwise Comparisons of Each Stratum in the New Staging Model Using an External Validation Cohort

This supplemental material has been provided by the authors to give readers additional information about their work.

© 2021 Waninger JJ et al. *JAMA Network Open.*

eTable 1. OS and PFS Measures by AJCC Eighth Edition M category

	Subjects alive at 1 year (%)	Median Survival (months)	D-Stat (95% CI)
**OS**			
M1a	91.9	not reached	1.4 (1.1, 1.8)
M1b	76	43.7	
M1c	65	43.9	
M1d	64	32.9	
**PFS**			
M1a	66.6	25.5	1.3 (1.0, 1.6)
M1b	54.4	23.6	
M1c	44.6	9.1	
M1d	45.2	8.3	

eTable 1. Percent of patients in each AJCC 8th edition M category alive (OS) or progressed (PFS) at 1 year and median survival time in months. Royston D statistic (D-Stat) of M staging model determined at the start of immunotherapy (ICB). M1a, patients with non-regional lymph nodes and/or skin/soft tissue lesions; M1b, patients with lung metastases; M1c, patients with all other visceral sites of disease (including liver involvement); M1d, patients with CNS metastases.

© 2021 Waninger JJ et al. *JAMA Network Open.*

eTable 2. Pairwise Comparisons of each AJCC Eighth Edition M category

	OS			PFS		
	**HR**	**95% CI**	**p-value**	**HR**	**95% CI**	**p-value**
M1a vs M1b	0.58	[0.30, 1.14]	0.11	0.79	[0.46, 1.36]	0.39
M1a vs M1c	0.57	[0.35, 0.95]	0.03	0.66	[0.44, 1.01]	0.05
M1a vs M1d	0.53	[0.31, 0.91]	0.02	0.62	[0.40, 0.96]	0.03
M1b vs. M1c	0.79	[0.51, 1.22]	0.29	0.79	[0.54, 1.14]	0.21
M1b vs. M1d	0.76	[0.48, 1.22]	0.26	0.74	[0.50, 1.11]	0.14
M1c vs. M1d	0.91	[0.64, 1.30]	0.61	0.92	[0.68, 1.24]	0.57

eTable 2. Pairwise comparisons of each AJCC 8th edition M category. HR, hazard ratio. CI, confidence interval. M1a, patients with non-regional lymph nodes and/or skin/soft tissue lesions; M1b, patients with lung metastases; M1c, patients with all other visceral sites of disease (including liver involvement); M1d, patients with CNS metastases.

© 2021 Waninger JJ et al. *JAMA Network Open.*

eTable 3. Royston D Statistic of M Staging Models

Endpoint	M stage	Royston D-Statistic	95% CI
OS	ICB	1.4	(1.1 ,1.8)
OS	Met Dx	1.3	(0.99 ,1.7)
OS	New	2.4	(1.8 ,3.2)
PFS	ICB	1.3	(1.0 ,1.6)
PFS	Met Dx	1.2	(0.97 ,1.5)
PFS	New	1.7	(1.3 ,2.1)

eTable 3. Royston D statistic of AJCC 8th edition M stage 1) determined at the start of immunotherapy (ICB), 2) at metastatic diagnosis (Met Dx), and 3) the newly proposed staging model at the start of immunotherapy (New) for both OS and PFS.

© 2021 Waninger JJ et al. *JAMA Network Open.*

eFigure 1. Kaplan-Meier Analysis of the OS and PFS in Patients With and Without Brain Metastases

A B

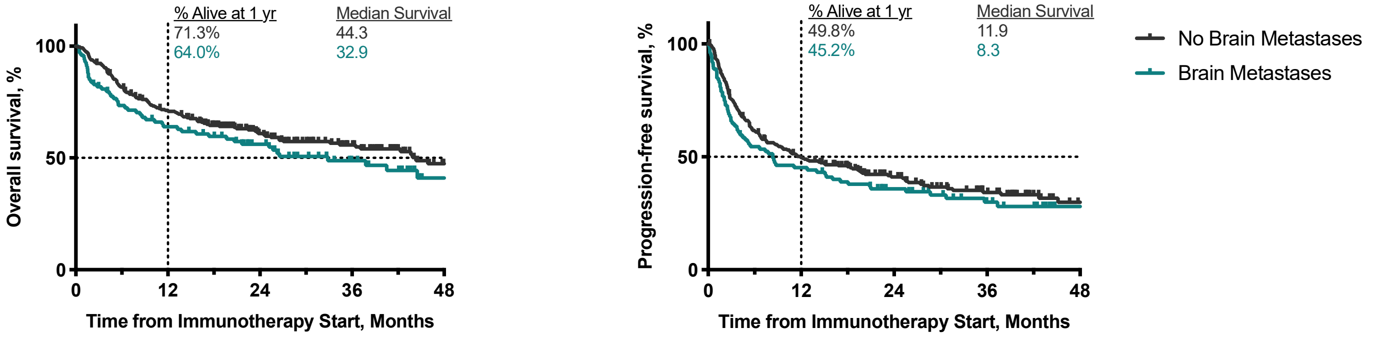

A

No. at risk									
No Brain Metastases	258	209	176	144	107	85	67	46	31
Brain Metastases	99	71	61	55	44	33	25	21	10

	Subjects, n (%)	HR (95% CI)	p-value
No Brain Metastases	258 (72.1)	0.79 (0.56, 1.12)	0.18
Brain Metastases	99 (27.7)		

B

No. at risk									
No Brain Metastases	258	158	124	101	70	53	39	24	15
Brain Metastases	99	54	45	38	30	24	18	15	7

	Subjects, n (%)	HR (95% CI)	p-value
No Brain Metastases	258 (72.1)	0.81 (0.60, 1.09)	0.16
Brain Metastases	99 (27.7)		

eFigure 1. Kaplan-Meier estimates of **A)** OS and **B)** PFS of patients with and without brain metastases. HR, hazard ratio; CI, confidence interval. Vertical dotted line marks 1-year survival.

© 2021 Waninger JJ et al. *JAMA Network Open.*

eFigure 2. Kaplan-Meier Analysis of AJCC Eighth Edition M Category at ICI Initiation in Patients Who Received First-Line ICI

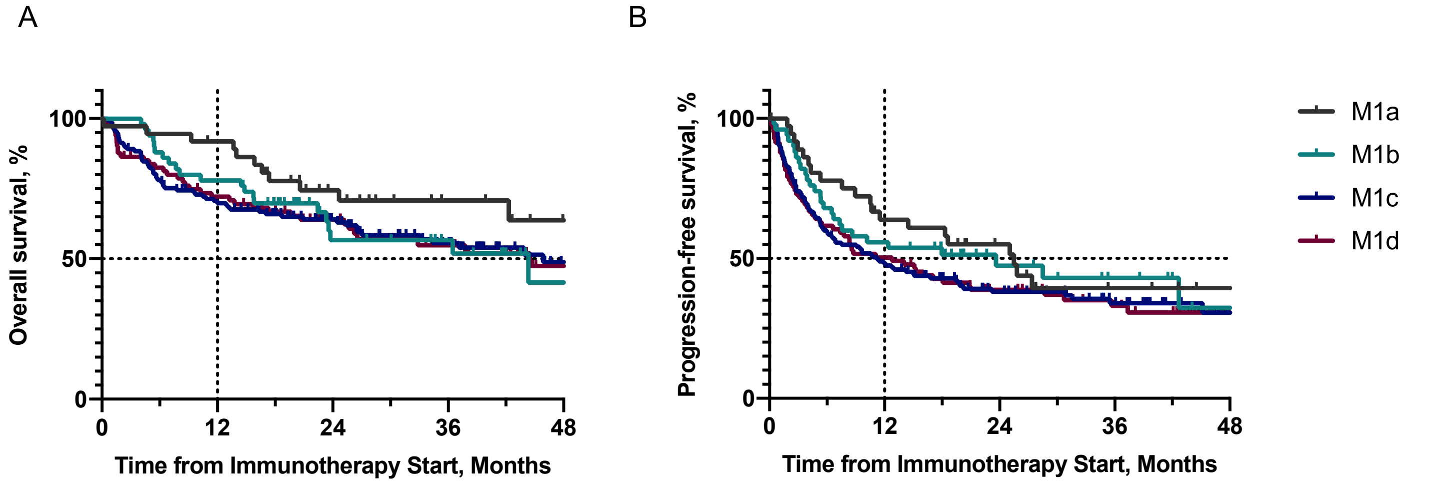

A — No. at risk

	0	6	12	18	24	30	36	42	48
M1a:	37	36	34	27	21	16	13	11	7
M1b:	50	45	40	32	18	17	13	9	5
M1c:	138	108	93	79	62	47	37	23	17
M1d:	81	65	56	50	41	30	22	19	9

B — No. at risk

	0	6	12	18	24	30	36	42	48
M1a:	37	29	23	22	16	9	8	6	4
M1b:	50	35	28	22	13	11	8	6	3
M1c:	138	83	66	51	37	32	23	12	8
M1d:	81	50	41	34	27	22	16	13	6

eFigure 2. Kaplan-Meier estimates of A) OS and B) PFS stratified by the AJCC 8th edition M category at ICI initiation for patients who received first line ICI. M1a, patients with non-regional lymph nodes and/or skin/soft tissue lesions; M1b, patients with lung metastases; M1c, patients with all other visceral sites of disease (including liver involvement); M1d, patients with CNS metastases. Vertical dotted line marks 1-year survival.

© 2021 Waninger JJ et al. *JAMA Network Open.*

eTable 4. Pairwise Comparisons of Each AJCC Eighth Edition M Category for Patients Who Received First-Line ICI

	OS			PFS		
	**HR**	**95% CI**	**p-value**	**HR**	**95% CI**	**p-value**
M1a vs M1b	0.6	[0.30, 1.20]	0.15	0.88	[0.49, 1.56]	0.66
M1a vs M1c	0.58	[0.34, 0.99]	0.05	0.72	[0.47, 1.12]	0.15
M1a vs M1d	0.6	[0.33, 1.10]	0.1	0.7	[0.43, 1.13]	0.14
M1b vs. M1c	0.84	[0.52, 1.35]	0.46	0.78	[0.52, 1.18]	0.25
M1b vs. M1d	0.92	[0.54, 1.57]	0.75	0.77	[0.50, 1.20]	0.25
M1c vs. M1d	1.04	[0.69, 1.56]	0.87	0.97	[0.69, 1.36]	0.85

eTable 4. Pairwise comparisons of each M category for patients who received first line ICI. M1a, patients with non-regional lymph nodes and/or skin/soft tissue lesions; M1b, patients with lung metastases; M1c, patients with all other visceral sites of disease (including liver involvement); M1d, patients with CNS metastases. HR, hazard ratio. CI, confidence interval.

© 2021 Waninger JJ et al. *JAMA Network Open.*

eFigure 3. Kaplan-Meier Analysis of Patient OS and PFS by *BRAF* Status

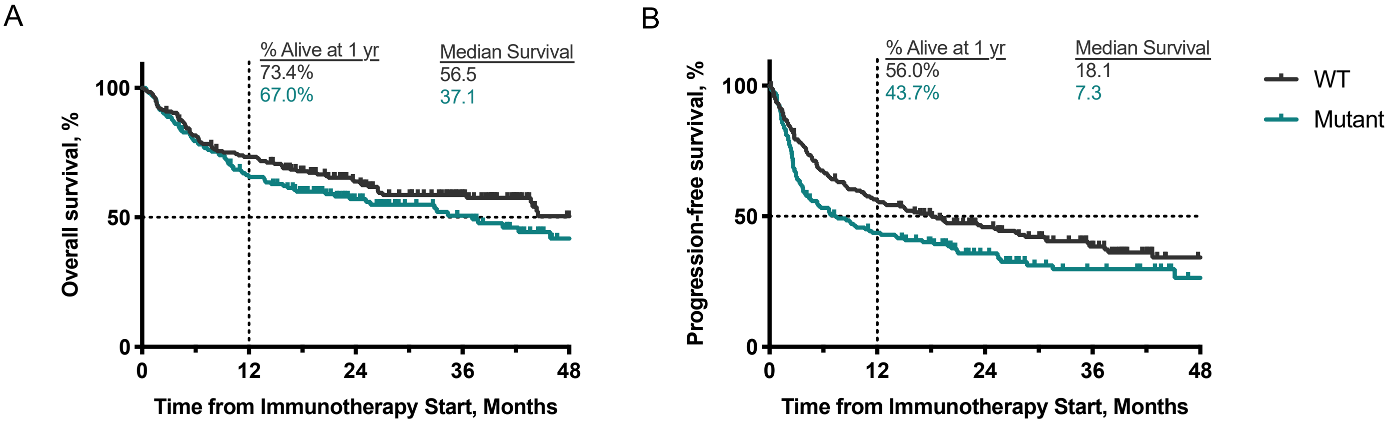

A)

No. at risk

WT	186	152	134	115	91	75	57	41	26
Mutant	152	119	95	79	56	42	36	27	16

	**Subjects, n (%)**	**HR (95% CI)**	**p-value**
WT	186 (55.0)	0.79 (0.57, 1.09)	0.15
Mutant	152 (45.0)		

B)

No. at risk

WT	186	125	103	85	65	54	38	23	14
Mutant	152	80	64	54	35	23	19	15	8

	**Subjects, n (%)**	**HR (95% CI)**	**p-value**
WT	186 (55.0)	0.73 (0.55, 0.96)	0.023
Mutant	152 (45.0)		

eFigure 3. Kaplan-Meier estimates of patient **A)** OS and **B)** PFS by BRAF mutation status. HR, hazard ratio; CI, confidence interval. Vertical dotted line marks 1-year survival.

© 2021 Waninger JJ et al. *JAMA Network Open.*

eFigure 4. Kaplan-Meier Analysis Comparing the OS and PFS of Patients With and Without Liver Metastases

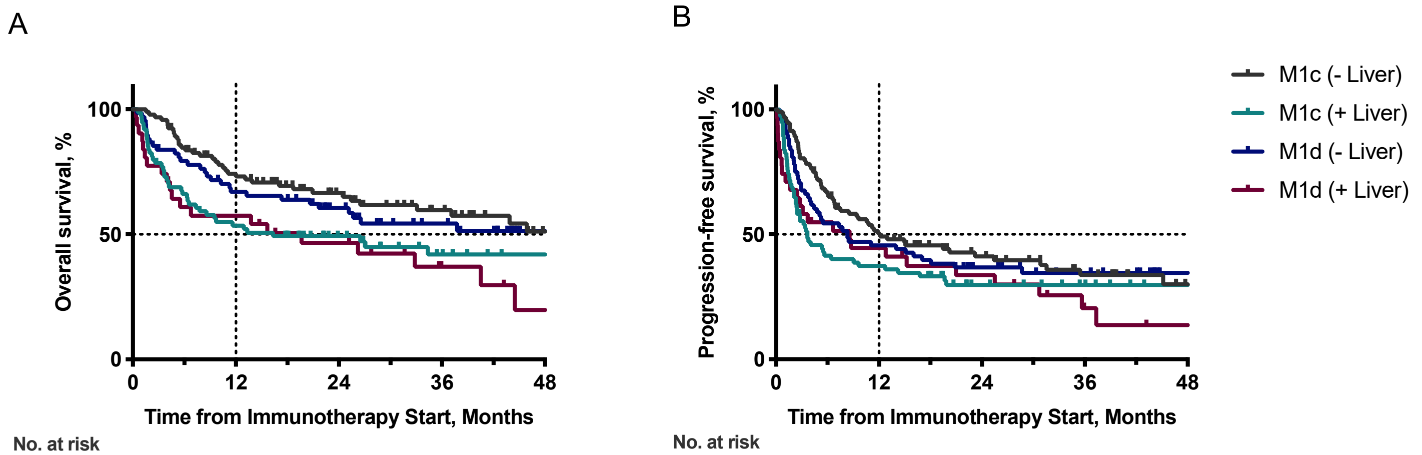

A) No. at risk

M1c (-Liver):	92	78	63	52	44	35	29	19	14
M1c (+Liver):	74	49	39	34	25	18	14	9	8
M1d (-Liver):	68	53	44	42	32	24	20	17	8
M1d (+Liver):	31	19	18	14	13	10	6	5	3

B) No. at risk

M1c (-Liver):	92	61	45	34	28	23	17	10	7
M1c (+Liver):	74	31	28	23	14	12	9	5	4
M1d (-Liver):	68	38	32	28	21	17	15	13	6
M1d (+Liver):	31	17	14	11	10	8	4	3	2

eFigure 4**.** Kaplan-Meier estimates of **A)** OS and **B)** PFS of M1c and M1d cutaneous melanoma patients with and without liver metastases. M1c (- Liver), patients in the M1c category without liver metastases; M1c (+Liver), patients in the M1c category with liver metastases; M1d (-Liver), patients with CNS metastases that do not have liver involvement; M1d (+ Liver), patients with both CNS and liver metastases; HR, hazard ratio; CI, confidence interval. Vertical dotted line marks 1-year survival.

© 2021 Waninger JJ et al. *JAMA Network Open.*

eTable 5. OS and PFS Measures for Patients With M1c and M1d Disease With and Without Liver Metastases

	Subjects alive at 1 year (%)	Median Survival (months)
**OS**		
M1c (-Liver)	73.1	56.5
M1c (+Liver)	54.9	16.3
M1d (-Liver)	66.9	62.0
M1d (+ Liver)	57.5	19.6
**PFS**		
M1c (-Liver)	50.3	12.1
M1c (+Liver)	37.4	3.7
M1d (-Liver)	45.6	8.3
M1d (+ Liver)	44.6	8.6

Extended Table 5. Percent of M1c or M1d patients with and without liver metastases alive (OS) or progressed (PFS) at 1 year and median survival time in months. M1c (- Liver), patients in the M1c category without liver metastases; M1c (+Liver), patients in the M1c category with liver metastases; M1d (-Liver), patients with CNS metastases that do not have liver involvement; M1d (+ Liver), patients with both CNS and liver metastases

© 2021 Waninger JJ et al. *JAMA Network Open.*

eTable 6. Pairwise Comparisons of Patients With M1c and M1d Disease With and Without Liver Metastases

	OS			PFS		
	**HR**	**95% CI**	**p-value**	**HR**	**95% CI**	**p-value**
M1c (-Liver) vs M1c (+Liver)	0.52	[0.33-0.82]	0.0043	0.64	[0.43-0.95]	0.025
M1c (- Liver) vs M1d (-Liver)	0.86	[0.52-1.36]	0.47	0.83	[0.56-1.23]	0.36
M1c (+ Liver) vs M1d (- Liver)	1.54	[0.97-2.45]	0.065	1.28	[0.86-1.91]	0.23
M1c (+Liver) vs M1d (+ Liver)	0.81	[0.47-1.43]	0.46	0.92	[0.56-1.51]	0.74
M1d (+Liver) vs M1d (-Liver)	0.53	[0.28-0.99]	0.045	0.71	[0.42-1.21]	0.21

Extended Table 6. Pairwise comparisons of M1c and M1d patients with and without liver metastases. M1c (-Liver), patients in the M1c category without liver metastases; M1c (+Liver), patients in the M1c category with liver metastases; M1d (-Liver), patients with CNS metastases that do not have liver involvement; M1d (+ Liver), patients with both CNS and liver metastases; HR, hazard ratio. CI, confidence interval.

© 2021 Waninger JJ et al. *JAMA Network Open.*

eFigure 5. Comparison of Number of Metastatic Sites and LDH Level

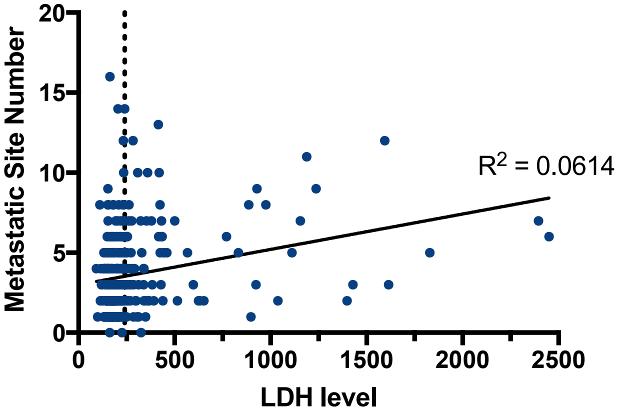

eFigure 5. Scatter plot of metastatic site number by LDH level. Dotted line is at 240 IU/L which is the upper limit of normal.

© 2021 Waninger JJ et al. *JAMA Network Open.*

eTable 7. Pairwise Univariable Analysis of LDH Stratified by Liver Involvement

**LDH at Metastatic Diagnosis**	**Presence of Liver Metastases**	**OS (months)**		**PFS (months)**	
		**HR [95% CI]**	**p-value**	**HR [95% CI]**	**p-value**
WNL	No	0.65 [0.39-1.10]	0.096	0.77 [0.51-1.18]	0.23
	Yes				
Elevated (> 240 IU/L)	No	0.42 [0.24-0.71]	0.0014	0.56 [0.34-0.92]	0.021
	Yes				
WNL	No	0.56 [0.33-0.95]	0.032	0.79 [0.52-1.21]	0.28
Elevated					
WNL	Yes	0.32 [0.18-0.56]	<0.0001	0.56 [0.34-0.91]	0.020
Elevated					

eTable 7. Pairwise comparisons of LDH level stratified by liver involvement. LDH, lactate dehydrogenase; WNL, within normal limits, ≤240 IU/L, elevated, >240 IU/L; HR, hazard ratio; CI, confidence interval.

© 2021 Waninger JJ et al. *JAMA Network Open.*

eTable 8. OS and PFS Measures of Patients in the Proposed Staging Model

	**Subjects alive at 1 year (%)**	**Median Survival (months)**
**OS**		
-Liver, LDH (WNL)	79.8	56.5
+Liver OR LDH (Elevated)	63.6	34.4
+Liver AND LDH (Elevated)	34.7	4.2
**PFS**		
-Liver, LDH (WNL)	55.0	18
+Liver OR LDH (Elevated)	43.7	8.5
+Liver AND LDH (Elevated)	33.2	2.1

eTable 8. Percent of patients alive at 1 year and median survival in each stratum of the new staging model. - Liver, all patients without liver metastases; + Liver, all patients with liver metastases; LDH, lactate dehydrogenase; WNL, within normal limits, ≤240 IU/L, elevated, >240 IU/L.

© 2021 Waninger JJ et al. *JAMA Network Open.*

eTable 9. Pairwise Comparisons of Each Stratum in the Proposed Staging Model of Patients With Metastatic Cutaneous Melanoma

	OS			PFS		
	**HR**	**95% CI**	**p-value**	**HR**	**95% CI**	**p-value**
+Liver OR LDH (Elevated) vs -Liver, LDH (WNL)	1.79	[1.19-2.67]	0.0049	1.32	[0.95-1.85]	0.10
+Liver AND LDH (Elevated) vs -Liver, LDH (WNL)	9.98	[5.30-18.77]	<0.0001	3.19	[1.93-5.26]	<0.0001
+Liver AND LDH (Elevated) vs +Liver OR LDH (Elevated)	3.01	[1.77-5.12]	<0.0001	1.85	[1.16-2.96]	0.0095

eTable 9**.** Pairwise comparisons of each stratum. 1) patients with normal LDH levels and no liver metastases (-Liver); 2), patients with either elevated LDH OR liver metastases (+Liver); 3), patients with both elevated LDH and liver metastases. - Liver, all patients without liver metastases; + Liver, all patients with liver metastases; LDH, lactate dehydrogenase; WNL, within normal limits, ≤240 IU/l, High, >240 IU/L; CI, confidence interval.

© 2021 Waninger JJ et al. *JAMA Network Open.*

eFigure 6. Kaplan-Meier Analysis of Progression-Free Survival of the Proposed Staging Model of Patients With Metastatic Cutaneous Melanoma Incorporating LDH Levels and Liver Involvement

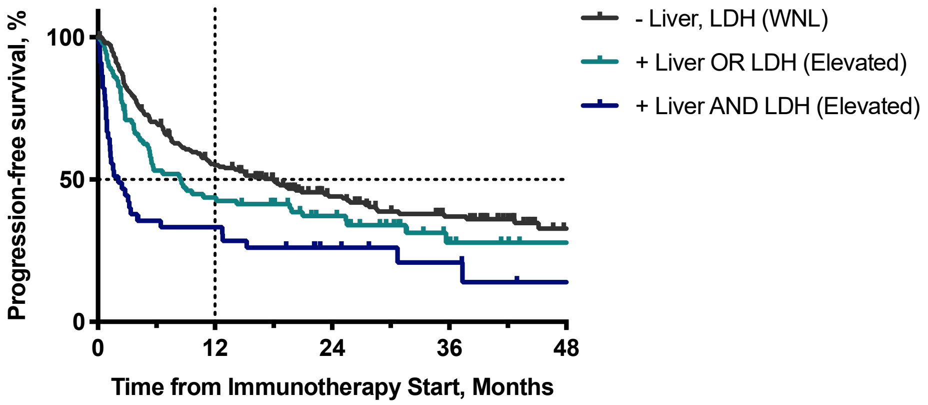

No. at risk

-Liver, LDH (WNL):	203	141	108	87	62	49	41	29	16
+Liver OR LDH (Elevated):	86	46	38	33	25	17	9	6	4
+Liver AND LDH (Elevated):	45	16	15	12	8	6	5	3	2

eFigure 6. Kaplan-Meier estimates of patient PFS using the proposed staging system for metastatic melanoma. LDH, lactate dehydrogenase; WNL, within normal limits, ≤240 IU/l, High, >240 IU/L; - Liver, all patients without liver metastases; + Liver, all patients with liver metastases; CI, confidence interval; vertical dotted line marks 1-year survival.

© 2021 Waninger JJ et al. *JAMA Network Open.*

eTable 10. Clinical and Disease Characteristics of External Validation Cohort

	**Factor**	**n (%)**	
	**Overall (n = 652)**		
	**Age at ICB Start**		
	Mean (SD)	67.9 (11.6)	
	Median (IQR)	68.6 (5.3)	
	**Gender**		
	Female	22 (3.4)	
	Male	630 (96.6)	
	**BRAF**		
	WT	256 (39.3)	
	MT	179 (27.5)	
	Unknown	217 (33.2)	
	**ECOG**		
	0	178 (27.3)	
	1	85 (13.0)	
	2+	17 (2.6)	
	Unknown	372 (57.1)	
	**ICB Type**		
	Dual Agent (Ipilimumab, Nivolumab)	89 (13.7)	
	Single Agent	563 (86.3)	
	**Presence of Liver Metastases**		
	No	503 (77.1)	
	Yes	149 (22.9)	
	**LDH**		
	Elevated	203 (31.1)	
	WNL	449 (68.9)	

© 2021 Waninger JJ et al. *JAMA Network Open.*

eTable 11. OS Measures of External Validation Cohort

	**Subjects alive at 1 year (%)**	**Median Survival (months)**
-Liver, LDH (WNL)	70.1	30.7
+Liver OR LDH (Elevated)	50.6	12.4
+Liver AND LDH (Elevated)	16.9	2.5

eTable 11. OS measures of external validation cohort. - Liver, all patients without liver metastases; + Liver, all patients with liver metastases LDH, lactate dehydrogenase; WNL, within normal limits.

© 2021 Waninger JJ et al. *JAMA Network Open.*

eTable 12. Pairwise Comparisons of Each Stratum in the New Staging Model Using an External Validation Cohort

	OS		
	HR	95% CI	p-value
+Liver OR LDH (Elevated) vs -Liver, LDH (WNL)	1.77	[1.40-2.24]	<0.0001
+Liver AND LDH (Elevated) vs -Liver, LDH (WNL)	37.65	[22.69-62.46]	<0.0001
+Liver AND LDH (Elevated) vs +Liver OR LDH (Elevated)	3.96	[2.72-5.78]	<0.0001

eTable 12. Pairwise comparisons of each stratum using a validation cohort. - Liver, all patients without liver metastases; + Liver, all patients with liver metastases; LDH, lactate dehydrogenase; WNL, within normal limits; HR, hazard ratio; CI, confidence interval.

© 2021 Waninger JJ et al. *JAMA Network Open.*
